# Inpatient Q Fever Frequency Is on the Rise

**DOI:** 10.1155/2023/4243312

**Published:** 2023-12-31

**Authors:** Mohamad Alhoda Mohamad Alahmad, Kassem A. Hammoud

**Affiliations:** ^1^University of Kansas Medical Center, Department of General Internal Medicine, Kansas City, KS, USA; ^2^University of Kansas Medical Center, Division of Infectious Diseases, Kansas City, KS, USA

## Abstract

**Background:**

Q fever is a zoonotic bacterial infection caused by Coxiella burnetii that is reportable in the USA. This infection is often asymptomatic; acute infection usually manifests as a self-limited febrile illness, hepatitis, or pneumonia. Chronic infection (usually infective endocarditis) often affects patients with valvulopathy or immunosuppression. Herein, we study the inpatient frequency of Q fever in the United States.

**Methods:**

We used a nationwide inpatient sample (NIS) for our retrospective cohort study to include hospitalizations with a diagnosis of Q fever between 2010 and 2019. Survey procedures were applied to accommodate for complex sampling design of NIS. Chi-square and least-square means were used for categorical and continuous variables, respectively. Jonckheere–Terpstra test was used to study the trends over the years. SAS 9.4 was used for data mining and analysis.

**Results:**

A total of 2,685 hospitalizations with a diagnosis of Q fever were included, among which 451 (17%) cases had a concurrent diagnosis of infective endocarditis. The mean age of patients was 58 years, and less than a third was female. Our analysis demonstrated that infective endocarditis was the most common cardiac complication associated with Q fever and was associated with increased inpatient mortality (*p* value <0.001). There is a trend of an increase in cases of inpatient Q fever with or without endocarditis over the years (*p* value <0.05). Q fever cases were more common across the Pacific and the South Atlantic divisions.

**Conclusion:**

Physicians should be aware of an increasing trend of hospitalized patients with Q fever and the significant association with infective endocarditis. Further studies are needed.

## 1. Introduction

Coxiella *burnetii* is an obligate intracellular, Gram-negative bacterium that causes a zoonosis called Q fever, which was first described in Australia in 1937. Q fever is usually diagnosed by serology [1], and it can present as an acute or chronic infection. The most common clinical manifestation of acute infection is a nonspecific febrile illness that might occur in conjunction with pneumonia or hepatitis. Chronic Q fever is rare, occurs in <5% of persons with acute infection [2], and includes endocarditis, chronic hepatitis, chronic vascular infections, osteomyelitis, osteoarthritis, and chronic pulmonary infections. Myocarditis and pericarditis have been reported although rare with this illness. Overall, this zoonotic disease is spread worldwide, and it is rare in the United States. It is classified as a potential bioterrorism agent by the Centers of Disease Control and Prevention (CDC), and it was designated as a nationally reportable disease in the United States in 1999. In 2008, the Q fever definition was changed to allow for the reporting of chronic and acute Q fever separately. The number of Q fever reported to CDC in 2000 was 19 cases. This number increased to 212 in 2019 (178 cases of acute Q fever and 34 cases of chronic Q fever) [3, 4]. Herein, we study the frequency of Q fever in hospital settings in the United States between 2010 and 2019.

## 2. Methodology and Material

The Department of Health and Human Services in the States has established a federal agency for research and quality improvement, the Agency for Healthcare Research and Quality (AHRQ). The latter has sponsored healthcare cost and the utilization project (HCUP) which includes multiple inpatient and outpatient databases. These databases do not include physician office visits and pharmacy or laboratory or radiology information. The project includes collaborative efforts between several states, the federal government, and the health industry. Inpatient databases include state inpatient databases (SID) which contain encounter abstracts of admissions from participating states and form the cornerstone for the National Inpatient Sample (NIS) which is a 20% stratified sample of all discharges from United States community hospitals that allow researchers to generate national and regional estimates of inpatient visits. For our research purposes, we utilized NIS databases for the years 2010–2019. We did not include the years after 2019 as the admission rate could have been affected by the coronavirus disease of 2019 (COVID-19) pandemic. We utilized the ICD-9 and the ICD-10 codes for Q fever (“0830” and “A78,” respectively) to define the cases for our project.

There was a change in ICD codes in 2015. The reports in the first three quarters of the year 2015 used ICD-9. The third quarter was reported using ICD-10 codes (see Supplementary Tables 1-2 for case definitions).

For NIS data after 2011, hospital ID was renamed to hospital NIS (HOSPID to HOSP_NIS). Also, the hospital division variable (HOSP_DIVISION) was not available before 2012. Instead, we were able to drive the value for hospital division from the hospital state variable (HOSPST) in the hospital NIS data file. In addition, in the year 2012, there was a change in the name of PL_NCHS2006 to PL_NCHS [5].

We included all encounters that contain a diagnosis of Q fever. Subpopulation analysis (domain analysis) was performed as per HCUP guidelines [6].

**Figure 1:**
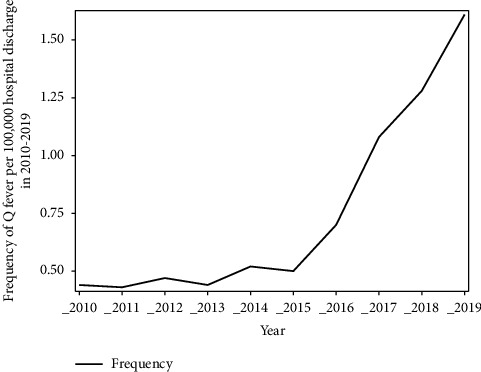
Frequency of inpatient Q fever.

**Figure 2:**
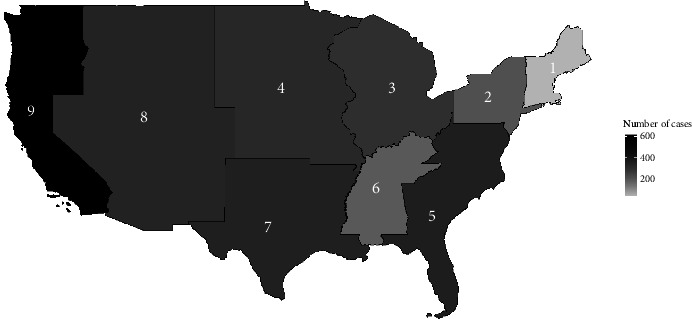
Frequency of inpatient Q fever by division in 2010−2019. Data source: HCUP-US.ahrq.gov, Nationwide Inpatient Sample, 2010−2019. 1, New England; 2, Middle Atlantic; 3, East North Central; 4, West North Central; 5, South Atlantic; 6, East South Central; 7, West South Central; 8, Mountain; 9, Pacific.

Utilizing the “SURVEY” procedure in Statistical Analysis Software (SAS) version 9.4, we obtained national estimates adjusting for complex data design. We compared the basic demographic information including gender, age, race, and socioeconomic status between cases that had a diagnosis of infective endocarditis and cases without this diagnosis, and we did not include other baseline variables that did not reach a statistical significance (diabetes and chronic liver disease). Comparison between different variables was evaluated using the appropriate test (Chi-square for categorical variables and least-square means for continuous variables). Logistic regression was used to study factors affecting inpatient mortality. Jonckheere–Terpstra test was used to study the trends over the years. The significance of 0.05 was used in all statistical analyses. A geometric map was carried out using R version 4.2.1 (Figure 1).  Figure 2 was created using JMP® Pro 17.0.0.

## 3. Results

This cohort included 2685 inpatient visits with a diagnosis of Q fever, of which 451 visits had a diagnosis of endocarditis. The mean age of hospitalized patients with a diagnosis of Q fever in 2010–2019 was around 58 years. The majority were Caucasian (63%) or Hispanic (15%). Only 29% were female. Compared to patients without endocarditis, cases of Q fever associated with infective endocarditis were older (mean age of 62 years versus 57 years, *p* value <0.001). They had a higher percentage of males (76% versus 70%, *p* value 0.1). Also, they were more likely to have heart failure, pulmonary hypertension, and valvular heart disease (*p* value <0.001) in comparison to patients without a concurrent diagnosis of infective endocarditis (Table 1).

**Figure 3:**
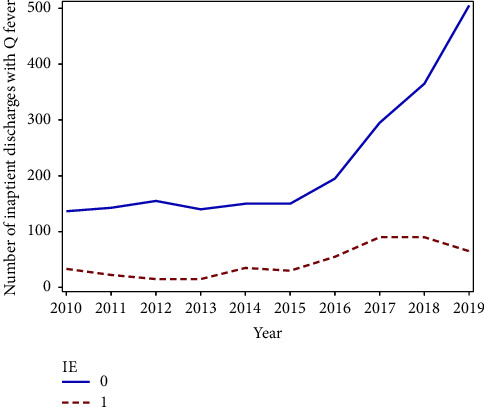
Inpatient Q fever trend with and without IE.

The main finding of this study is the increase in inpatient frequency of cases with Q fever especially since 2013.  Figure 3 shows an upward trend in the number of discharged cases with a diagnosis of Q fever with infective endocarditis (dotted red line) and without discharge diagnosis of endocarditis (solid blue line) across the decade from 2010 to 2019. The frequency of Q fever per 100,000 hospital discharges each year across the years from 2010 to 2019 has increased as well (see Figure 1). This upward trend is primarily driven by an increase in frequency in East North Central, West North Central, South Atlantic, West South Central, and Pacific divisions (see Figure 2).  Figure 2 shows the density of hospital discharges with a Q fever from 2010 to 2019 across each United States division. It indicates that Q fever cases were more frequent across the Pacific and the South Atlantic divisions.

Table 2 presents patients' outcomes during hospital stay. Most of the cases were admitted to hospitals in large metropolitan areas (more than 46% of the cases). The length of the stay was on average 12 days. Inpatient mortality overall was high (4%) in comparison to average inpatient mortality of 2% [7]. Patients with Q fever and infective endocarditis had higher inpatient mortality (7% versus 3%, *p* value 0.01). Most cases were discharged home although a higher percentage of people with endocarditis was discharged home with home healthcare or to a facility.

## 4. Discussion

In this study, we report an upward trend of inpatient Q fever frequency from 0.4 per 100,000 discharges in 2010 to 1.6 per 100,000 discharges in 2019. Although Q fever frequency and incidence are historically underestimated [8], our findings correlate with the increased number of annually reported cases to the Centers for Disease Control and Prevention (CDC) from 135 reported cases in 2010 to 212 cases in 2019 [9].

Q fever is a rare but potentially fatal disease. Its occurrence is usually sporadic although outbreak of hundreds of cases has been reported [10]. *C. burnetii* is very resistant to physical stresses, including heat and desiccation, and can survive in the environment for months to years. It is transmitted mainly via aerosols, and the most common animal reservoirs are cattle, sheep, and goats. Hence, selective vaccination is the best strategy for prevention in humans as elimination is difficult [11].

Before 1989, only 234 cases had been reported in the literature [12]. The incidence of Q fever, although still rare, has doubled in the US from 2008 to 2017 [13]. Our study shows that inpatient frequency is also trending up between 2010 and 2019. A Mayo Clinic study, conducted from 1980 to 2005, declared the absence of reliable reporting for Q fever endocarditis [14]. Cases and outbreaks of Q fever have increased in France from 1985 to 2009 [15].

Some patients (5–19%) with Q fever can develop IE [8, 16–18]. Q fever endocarditis was almost unknown in the United States before 1979 [19]. Positive anticardiolipin antibodies (>22 immunoglobulin G-type phospholipid units (GPLU)) were independently associated with acute Q fever endocarditis (odds ratio (OR): 2.7, 95% confidence interval [1.3-5.5], and *p* value =0.004) [20]. Frequency of complications with chronic Q fever is up to 34%, and they are adverse prognosticators [21]. Valvulopathy is a risk factor especially aortic and mitral regurgitation [22]. Development of arterial fistula has been associated with high mortality rates [23]. A phase I IgG antibody titer >1: 800 or a single positive blood culture for C. burnetii serves as a major criterion for infective endocarditis [22]. A review of Q fever cases submitted between 1999 and 2015 suggests that phase I and phase II titers comparisons may need to be reexamined as some cases were not classifiable by the criteria of the Council for State and Territorial Epidemiologists (CSTE) [22].

Our study shows that patients with Q fever and endocarditis compared to cases without endocarditis tend to be associated significantly with valvulopathy (81% vs. 13%), heart failure (52% vs. 20%), coronary artery disease (26% vs. 14%), and pulmonary hypertension (14% vs. 6%). This correlates with the literature that Q fever endocarditis usually affects patients with previous valvular disease [8].

In our study, a concurrent diagnosis of unspecified endocarditis was considered related to Q fever as there is no specific ICD-9 or ICD-10 code for Q fever-related endocarditis.

A small study suggests that acute Q fever-related myocarditis is rare (0.6%–3%) [24, 25]. In our study of 2,685 discharges that had Q fever, only thirty cases had either a concurrent diagnosis of myocarditis or pericarditis.

Although our study was not designed to study Q fever mortality, we noted that patients with Q fever and endocarditis had higher inpatient mortality compared to cases without concurrent diagnosis of endocarditis (7% vs. 3%, *p* value 0.01). Patients with acute Q fever should be carefully assessed for the risk of progression to chronic Q fever, and this includes a review of possible immunosuppression, pregnancy, and vascular and valvular heart disease. If they have any of the above risk factors, they should be monitored with a series of serologic and physical exams for at least 2 years [3].

Patients with chronic Q fever should be screened with an echocardiogram (preferably transesophageal) to look for endocarditis especially if they have a preexistent valvular disease. Positron emission tomographic (PET) scanning could be proposed for all patients with suspected persistent focused infection to rapidly diagnose vascular and lymphatic infections associated with death and lymphoma, respectively [26].

A similar national study in Spain revealed that Q fever is associated with high-cost long hospitalization. Also, patients with severe clinical presentations had higher mortality stressing the importance of early suspicion and detection [27]. Our study shows that cases with endocarditis had higher average charges ($151,378 vs. $127,307, *p* value <0.001).

Due to the retrospective design, the study has several limitations. First, there is no specific ICD code for acuity of Q fever infection or for Q fever complications such as endocarditis. Also, other complications or confounders may not have been captured in the study design. We cannot presume that the increased frequency of Q fever discharge diagnosis correlates with increased prevalence and/or incidence of the disease, and this trend is possibly related to increased awareness and testing for Q fever. Moreover, this trend could be a physician's response to CDC recommendations in 2023 [28]. Further studies are needed. Also, the NIS database provides abstracts at the encounter-level rather than patient-level. This means that infected patients with multiple admissions could be counted multiple times. Furthermore, the NIS data may not be necessarily an unbiased sample of Q fever patients. Also, it is hard to say if the primary reason for admission was related to Q fever. In addition, coding errors might happen which may affect results.

In conclusion, clinicians and public health experts should pay attention to the possible increasing frequency of Q fever especially across the Pacific and South Atlantic divisions. Clinicians should screen for endocarditis, especially in patients with valvular/vascular disease and/or immunosuppression. Early detection of Q fever and treatment is associated with better outcomes.

## Figures and Tables

**Table 1:** Baseline characteristics.

Variable	Total	With IE	Without IE	*p* value
Demographics				
Age mean (SD)	57.7 (40.4)	62.1 (33.6)	56.8 (41.4)	<0.001
Female gender	788 (29.3%)	110 (24.4%)	678 (30.3%)	0.1138
Race				
White	1689 (62.9%)	273 (60.5%)	1416 (63.4%)	0.2479
Black	189 (7.0%)	35 (7.8%)	154 (6.9%)	
Hispanic	406 (15.1%)	79 (17.6%)	326 (14.6%)	
Other	215 (8.0%)	20 (4.4%)	195 (8.7%)	
Comorbidities				
Transplant	85 (3.2%)	—	80 (3.6%)	<0.0001
Malignancy	344 (12.8%)	—	334 (14.9%)	<0.0001
PVD	303 (11.3%)	90 (20.0%)	213 (9.5%)	0.0015
Heart failure	680 (25.3%)	233 (51.7%)	447 (20.0%)	<0.0001
Pulmonary HTN	200 (7.5%)	64 (14.2%)	136 (6.1%)	0.0008
CKD	588 (21.9%)	143 (31.7%)	445 (19.9%)	0.002
Valvular disease	660 (24.6%)	367 (81.4%)	293 (13.1%)	<0.0001
Acute stroke	177 (6.6%)	59 (13.1%)	118 (5.3%)	0.0003
AMI	80 (3.0%)	30 (6.7%)	50 (2.2%)	0.0097
CAD	430 (16.0%)	115 (25.5%)	315 (14.1%)	0.0011
Hospital location				
Central metropolitan	790 (29.4%)	117 (26.0%)	673 (30.1%)	0.3039
Fringe metropolitan	444 (16.5%)	50 (11.1%)	394 (17.6%)	
Medium metropolitan	485 (18.1%)	94 (20.9%)	391 (17.5%)	
Small metropolitan	283 (10.5%)	50 (11.1%)	233 (10.4%)	
Micropolitan counties	317(11.8%)	55 (12.1%)	263 (11.8%)	
Other	346 (12.9%)	70 (15.5%)	276 (12.4%)	
Socioeconomic status				
0–25th percentile	763 (28.4%)	124 (27.4%)	639 (28.6%)	0.0768
26th–50th percentile (median)	718 (26.7%)	85 (18.9%)	633 (28.3%)	
51st–75th percentile	714 (26.6%)	147 (32.6%)	567 (25.4%)	
76th–100th percentile	436 (16.3%)	80 (17.7%)	356 (16.0%)	
Primary payor				
Medicare	1126 (42.0%)	219 (48.6%)	907 (40.6%)	<0.0001
Medicaid	440 (16.4%)	62 (13.9%)	378 (16.9%)	
Private	890 (33.1%)	99 (22.0%)	790 (35.4%)	
Other	229 (8.5%)	70 (15.6%)	159 (7.1%)	

AMI: acute myocardial infarction. CAD: coronary artery disease. CKD: chronic kidney disease. HTN: hypertension. PVD: peripheral vascular disease. SD: standard deviation.

**Table 2:** Outcomes.

Variable	Total	With IE	Without IE	*p* value
Index mortality, *n* (%)	108 (4.0%)	33 (7.3%)	74 (3.3%)	0.0144
LOS mean (SD), days	11.2 (42.8)	12.4 (27.9)	11.0 (45.2)	<0.0001
Total charges (SD), $	131,355 (589,516)	151,378 (412,632)	127,307 (619,104)	<0.0001
*Discharge disposition*				
Discharged to home	1639 (61.0%)	194 (43.1%)	1445 (64.7%)	<0.0001
Transferred	115 (4.3%)	45 (10.0%)	70 (3.1%)	
Discharged to facility	446 (16.6%)	118 (26.3%)	327 (14.6%)	
Home health care	368 (13.7%)	60 (13.3%)	308 (13.8%)	
Other	118 (4.4%)	33 (7.4%)	84 (3.8%)	

LOS: length of stay. SD: standard deviation.

## Data Availability

All data are available for the public on the HCUP website.

## Supplementary Materials

Supplementary Materials Supplementary Table 1: Q fever and cardiac complication definitions. Supplementary Table 2: ICD-10 codes for diagnosis used in study design.
