# Supplementary material for: Inpatient Q Fever Frequency Is on the Rise
Source: Can J Infect Dis Med Microbiol. 2023 Dec 31;2023:4243312. doi: 10.1155/2023/4243312 (PMC10771919; doi:10.1155/2023/4243312)
Supplement: Supplementary Materials — Supplementary Table 1: Q fever and cardiac complication definitions. Supplementary Table 2: ICD-10 codes for diagnosis used in study design. [file 4243312.f1.docx]

**Supplementary materials**

Supplementary Table 1 : Q fever and cardiac complications definitions

| ICD revision | Diagnosis | **Code** | **Description** |
| --- | --- | --- | --- |
| 9^th^ | Q fever | "0830", | Q fever |
|  | Pericarditis | "4200", | Acute pericarditis in diseases classified elsewhere |
|  |  | "42090", | Acute pericarditis, unspecified |
|  |  | "42099", | Other acute pericarditis |
|  | Endocarditis | "4210", | Acute and subacute bacterial endocarditis |
|  |  | "4211", | Acute and subacute infective endocarditis in diseases classified elsewhere |
|  |  | "4219", | Acute endocarditis, unspecified |
|  |  | "42490", | Endocarditis, valve unspecified, unspecified cause |
|  |  | "42491", | Endocarditis in diseases classified elsewhere |
|  |  | "42499", | Other endocarditis, valve unspecified |
|  | Myocarditis | "4220", | Acute myocarditis in diseases classified elsewhere |
|  |  | "42290", | Acute myocarditis, unspecified |
|  |  | "42299", | Other acute myocarditis |
|  |  | "4290", | Myocarditis, unspecified |
| 10^th^ | Q fever | "A78", | Q fever |
|  | Pericarditis | "I300", | Acute nonspecific idiopathic pericarditis |
|  |  | "I301", | Infective pericarditis |
|  |  | "I308", | Other forms of acute pericarditis |
|  |  | "I309", | Acute pericarditis, unspecified |
|  |  | "I310", | Chronic adhesive pericarditis |
|  |  | "I311", | Chronic constrictive pericarditis |
|  |  | "I318", | Other specified diseases of pericardium |
|  |  | "I319", | Disease of pericardium, unspecified |
|  |  | "I32", | Pericarditis in diseases classified elsewhere |
|  | Endocarditis | "I330", | Acute and subacute infective endocarditis |
|  |  | "I339", | Acute and subacute endocarditis, unspecified |
|  |  | "I38", | Endocarditis, valve unspecified |
|  |  | "I39", | Endocarditis and heart valve disorders in diseases classified elsewhere |
|  | Myocarditis | "I400", | Infective myocarditis |
|  |  | "I401", | Isolated myocarditis |
|  |  | "I408", | Other acute myocarditis |
|  |  | "I409", | Acute myocarditis, unspecified |
|  |  | "I41", | Myocarditis in diseases classified elsewhere |
|  |  | "I514", | Myocarditis, unspecified |

*ICD: International Classification of Diseases*

Supplementary table 2 : ICD-10 codes for diagnoses used in study design

| Case definition | ICD-10 Codes |
| --- | --- |
| Acute myocardial infarction | "I2101", "I2102", "I2109", "I2111", "I2119", "I2121", "I2129", "I213", "I214", "I219", "I21A1", "I21A9", "I220", "I221", "I222", "I228", "I229", "I230", "I231", "I232", "I233", "I234",  "I235", "I236", "I238" |
| Heart failure | "I0981", "I501", "I5020", "I5021", "I5022", "I5023", "I5030", "I5031", "I5032", "I5033", "I5040", "I5041", "I5042", "I5043", "I50810", "I50811", "I50812", "I50813", "I50814", "I5082", "I5083", "I5084", "I5089", "I509", "I5181", "I97130", "I97131", "O29121", "O29122", "O29123", "O29129",  "R570", "Z95811", "Z95812" |
| Coagulopathy | "D6109", "D611", "D612", "D613", "D61810", "D61811", "D61818", "D6182", "D6189", "D619", "D65", "D66", "D67", "D680", "D681", "D682", "D68311", "D68312", "D68318", "D6832", "D684", "D688", "D689", "D691", "D693", "D6941", "D6942", "D6949", "D6951", "D6959", "D696", "D698", "D699", "D7582", "O99111", "O99112", "O99113",  "O99119", "O9912", "O9913" |
| Chronic liver disease | "A5145", "A5274", "B180", "B181", "B182", "B188", "B189", "B1910", "B1920", "B199", "B251", "B581", "K700", "K702", "K7030", "K7031", "K709", "K713", "K714", "K7150", "K7151", "K716", "K717", "K718", "K730", "K731", "K732", "K738", "K739", "K740", "K7400", "K7401", "K7402", "K741", "K742", "K743", "K744", "K745", "K7460", "K7469", "K751", "K752", "K753", "K754", "K7581", "K7589", "K759", "K760", "K761", "K762", "K763", "K764", "K7681",  "K7689", "K769", "K77", "B190", "B1911", "B1921", "I8500", "I8501", "I8510", "I8511", "I864", "K7040", "K7041", "K7210", "K7211", "K7290", "K7291", "K765", "K766",  "K767", "K9182", "Z944" |
| Chronic kidney disease | "N184", "N185", "N186", "Z4901", "Z4902", "Z4931", "Z4932", "Z9115", "Z940", "Z992", "N183", "N1830", "N1831", "N1832", "N189", "N19" |
| Obesity | "E6601", "E6609", "E661", "E662", "E668", "E669", "O99210", "O99211", "O99212", "O99213", "O99214", "O99215", "R939", "Z6830", "Z6831", "Z6832", "Z6833", "Z6834", "Z6835", "Z6836", "Z6837", "Z6838", "Z6839", "Z6841", "Z6842", "Z6843",  "Z6844", "Z6845", "Z6854" |
| Malignancies |  |
| Metastatic disease | "C770", "C771", "C772", "C773", "C774", "C775", "C778", "C779", "C7800", "C7801", "C7802", "C781", "C782", "C7830", "C7839", "C784", "C785", "C786", "C787", "C7880", "C7889", "C7900", "C7901", "C7902", "C7910", "C7911", "C7919", "C792", "C7931", "C7932", "C7940", "C7949", "C7951", "C7952", "C7960", "C7961", "C7962", "C7970", "C7971", "C7972", "C7981", "C7982", "C7989", "C799", "C7B00", "C7B01", "C7B02", "C7B03", "C7B04", "C7B09", "C7B1", "C7B8", "C800" |
| Leukemias | "C9010", "C9011", "C9012", "C9100", "C9101", "C9102", "C9110", "C9111", "C9112", "C9130", "C9131", "C9132", "C9140", "C9141", "C9142", "C9150", "C9151", "C9152", "C9160", "C9161", "C9162", "C9190", "C9191", "C9192", "C91A0", "C91A1", "C91A2", "C91Z0", "C91Z1", "C91Z2", "C9200", "C9201", "C9202", "C9210", "C9211", "C9212", "C9220", "C9221", "C9222", "C9230", "C9231", "C9232", "C9240", "C9241", "C9242", "C9250", "C9251", "C9252", "C9260", "C9261", "C9262", "C9290", "C9291", "C9292", "C92A0", "C92A1", "C92A2", "C92Z0", "C92Z1", "C92Z2", "C9300", "C9301", "C9302", "C9310", "C9311", "C9312", "C9330", "C9331", "C9332", "C9390", "C9391", "C9392", "C93Z0", "C93Z1", "C93Z2", "C9400", "C9401", "C9402", "C9420", "C9421", "C9422", "C9430", "C9431", "C9432", "C9440", "C9441", "C9442", "C946", "C9480", "C9481", "C9482", "C9500", "C9501", "C9502", "C9510", "C9511", "C9512", "C9590", "C9591", "C9592" |
| Lymphoma | "C8100", "C8101", "C8102", "C8103", "C8104", "C8105", "C8106", "C8107", "C8108", "C8109", "C8110", "C8111", "C8112", "C8113", "C8114", "C8115", "C8116", "C8117", "C8118", "C8119", "C8120", "C8121", "C8122", "C8123", "C8124", "C8125", "C8126", "C8127", "C8128", "C8129", "C8130", "C8131", "C8132", "C8133", "C8134", "C8135", "C8136", "C8137", "C8138", "C8139", "C8140", "C8141", "C8142", "C8143", "C8144", "C8145", "C8146", "C8147", "C8148", "C8149", "C8170", "C8171", "C8172", "C8173", "C8174", "C8175", "C8176", "C8177", "C8178", "C8179", "C8190", "C8191", "C8192", "C8193", "C8194", "C8195", "C8196", "C8197", "C8198", "C8199", "C8200", "C8201", "C8202", "C8203", "C8204", "C8205", "C8206", "C8207", "C8208", "C8209", "C8210", "C8211", "C8212", "C8213", "C8214", "C8215", "C8216", "C8217", "C8218", "C8219", "C8220", "C8221", "C8222", "C8223", "C8224", "C8225", "C8226", "C8227", "C8228", "C8229", "C8230", "C8231", "C8232", "C8233", "C8234", "C8235", "C8236", "C8237", "C8238", "C8239", "C8240", "C8241", "C8242", "C8243", "C8244", "C8245", "C8246", "C8247", "C8248", "C8249", "C8250", "C8251", "C8252", "C8253", "C8254", "C8255", "C8256", "C8257", "C8258", "C8259", "C8260", "C8261", "C8262", "C8263", "C8264", "C8265", "C8266", "C8267", "C8268", "C8269", "C8280", "C8281", "C8282", "C8283", "C8284", "C8285", "C8286", "C8287", "C8288", "C8289", "C8290", "C8291", "C8292", "C8293", "C8294", "C8295", "C8296", "C8297", "C8298", "C8299", "C8300", "C8301", "C8302", "C8303", "C8304", "C8305", "C8306", "C8307", "C8308", "C8309", "C8310", "C8311", "C8312", "C8313", "C8314", "C8315", "C8316", "C8317", "C8318", "C8319", "C8330", "C8331", "C8332", "C8333", "C8334", "C8335", "C8336", "C8337", "C8338", "C8339", "C8350", "C8351", "C8352", "C8353", "C8354", "C8355", "C8356", "C8357", "C8358", "C8359", "C8370", "C8371", "C8372", "C8373", "C8374", "C8375", "C8376", "C8377", "C8378", "C8379", "C8380", "C8381", "C8382", "C8383", "C8384", "C8385", "C8386", "C8387", "C8388", "C8389", "C8390", "C8391", "C8392", "C8393", "C8394", "C8395", "C8396", "C8397", "C8398", "C8399", "C8400", "C8401", "C8402", "C8403", "C8404", "C8405", "C8406", "C8407", "C8408", "C8409", "C8410", "C8411", "C8412", "C8413", "C8414", "C8415", "C8416", "C8417", "C8418", "C8419", "C8440", "C8441", "C8442", "C8443", "C8444", "C8445", "C8446", "C8447", "C8448", "C8449", "C8460", "C8461", "C8462", "C8463", "C8464", "C8465", "C8466", "C8467", "C8468", "C8469", "C8470", "C8471", "C8472", "C8473", "C8474", "C8475", "C8476", "C8477", "C8478", "C8479", "C8490", "C8491", "C8492", "C8493", "C8494", "C8495", "C8496", "C8497", "C8498", "C8499", "C84A0", "C84A1", "C84A2", "C84A3", "C84A4", "C84A5", "C84A6", "C84A7", "C84A8", "C84A9", "C84Z0", "C84Z1", "C84Z2", "C84Z3", "C84Z4", "C84Z5", "C84Z6", "C84Z7", "C84Z8", "C84Z9", "C8510", "C8511", "C8512", "C8513", "C8514", "C8515", "C8516", "C8517", "C8518", "C8519", "C8520", "C8521", "C8522", "C8523", "C8524", "C8525", "C8526", "C8527", "C8528", "C8529", "C8580", "C8581", "C8582", "C8583", "C8584", "C8585", "C8586", "C8587", "C8588", "C8589", "C8590", "C8591", "C8592", "C8593", "C8594", "C8595", "C8596", "C8597", "C8598", "C8599", "C860", "C861", "C862", "C863", "C864", "C865", "C866", "C880", "C882", "C883", "C884", "C888", "C889", "C9000", "C9001", "C9002", "C9020", "C9021", "C9022", "C9030", "C9031", "C9032", "C960", "C962", "C9620", "C9621", "C9622", "C9629", "C964", "C969", "C96A", "C96Z", "D47Z9" |
| Solid cancers | "C000", "C001", "C002", "C003", "C004", "C005", "C006", "C008", "C009", "C01", "C020", "C021", "C022", "C023", "C024", "C028", "C029", "C030", "C031", "C039", "C040", "C041", "C048", "C049", "C050", "C051", "C052", "C058", "C059", "C060", "C061", "C062", "C0680", "C0689", "C069", "C07", "C080", "C081", "C089", "C090", "C091", "C098", "C099", "C100", "C101", "C102", "C103", "C104", "C108", "C109", "C110", "C111", "C112", "C113", "C118", "C119", "C12", "C130", "C131", "C132", "C138", "C139", "C140", "C142", "C148", "C153", "C154", "C155", "C158", "C159", "C160", "C161", "C162", "C163", "C164", "C165", "C166", "C168", "C169", "C170", "C171", "C172", "C173", "C178", "C179", "C180", "C181", "C182", "C183", "C184", "C185", "C186", "C187", "C188", "C189", "C19", "C20", "C210", "C211", "C212", "C218", "C220", "C221", "C222", "C223", "C224", "C227", "C228", "C229", "C23", "C240", "C241", "C248", "C249", "C250", "C251", "C252", "C253", "C254", "C257", "C258", "C259", "C260", "C261", "C269", "C300", "C301", "C310", "C311", "C312", "C313", "C318", "C319", "C320", "C321", "C322", "C323", "C328", "C329", "C33", "C3400", "C3401", "C3402", "C3410", "C3411", "C3412", "C342", "C3430", "C3431", "C3432", "C3480", "C3481", "C3482", "C3490", "C3491", "C3492", "C37", "C380", "C381", "C382", "C383", "C384", "C388", "C390", "C399", "C4000", "C4001", "C4002", "C4010", "C4011", "C4012", "C4020", "C4021", "C4022", "C4030", "C4031", "C4032", "C4080", "C4081", "C4082", "C4090", "C4091", "C4092", "C410", "C411", "C412", "C413", "C414", "C419", "C430", "C4310", "C4311", "C43111", "C43112", "C4312", "C43121", "C43122", "C4320", "C4321", "C4322", "C4330", "C4331", "C4339", "C434", "C4351", "C4352", "C4359", "C4360", "C4361", "C4362", "C4370", "C4371", "C4372", "C438", "C439", "C4400", "C4409", "C44101", "C44102", "C441021", "C441022", "C44109", "C441091", "C441092", "C44131", "C441321", "C441322", "C441391", "C441392", "C44191", "C44192", "C441921", "C441922", "C44199", "C441991", "C441992", "C44201", "C44202", "C44209", "C44291", "C44292", "C44299", "C44300", "C44301", "C44309", "C44390", "C44391", "C44399", "C4440", "C4449", "C44500", "C44501", "C44509", "C44590", "C44591", "C44599", "C44601", "C44602", "C44609", "C44691", "C44692", "C44699", "C44701", "C44702", "C44709", "C44791", "C44792", "C44799", "C4480", "C4489", "C4490", "C4499", "C450", "C451", "C452", "C457", "C459", "C460", "C461", "C462", "C463", "C464", "C4650", "C4651", "C4652", "C467", "C469", "C470", "C4710", "C4711", "C4712", "C4720", "C4721", "C4722", "C473", "C474", "C475", "C476", "C478", "C479", "C480", "C481", "C482", "C488", "C490", "C4910", "C4911", "C4912", "C4920", "C4921", "C4922", "C493", "C494", "C495", "C496", "C498", "C499", "C49A0", "C49A1", "C49A2", "C49A3", "C49A4", "C49A5", "C49A9", "C4A0", "C4A10", "C4A11", "C4A111", "C4A112", "C4A12", "C4A121", "C4A122", "C4A20", "C4A21", "C4A22", "C4A30", "C4A31", "C4A39", "C4A4", "C4A51", "C4A52", "C4A59", "C4A60", "C4A61", "C4A62", "C4A70", "C4A71", "C4A72", "C4A8", "C4A9", "C50011", "C50012", "C50019", "C50021", "C50022", "C50029", "C50111", "C50112", "C50119", "C50121", "C50122", "C50129", "C50211", "C50212", "C50219", "C50221", "C50222", "C50229", "C50311", "C50312", "C50319", "C50321", "C50322", "C50329", "C50411", "C50412", "C50419", "C50421", "C50422", "C50429", "C50511", "C50512", "C50519", "C50521", "C50522", "C50529", "C50611", "C50612", "C50619", "C50621", "C50622", "C50629", "C50811", "C50812", "C50819", "C50821", "C50822", "C50829", "C50911", "C50912", "C50919", "C50921", "C50922", "C50929", "C510", "C511", "C512", "C518", "C519", "C52", "C530", "C531", "C538", "C539", "C540", "C541", "C542", "C543", "C548", "C549", "C55", "C561", "C562", "C569", "C5700", "C5701", "C5702", "C5710", "C5711", "C5712", "C5720", "C5721", "C5722", "C573", "C574", "C577", "C578", "C579", "C58", "C600", "C601", "C602", "C608", "C609", "C61", "C6200", "C6201", "C6202", "C6210", "C6211", "C6212", "C6290", "C6291", "C6292", "C6300", "C6301", "C6302", "C6310", "C6311", "C6312", "C632", "C637", "C638", "C639", "C641", "C642", "C649", "C651", "C652", "C659", "C661", "C662", "C669", "C670", "C671", "C672", "C673", "C674", "C675", "C676", "C677", "C678", "C679", "C680", "C681", "C688", "C689", "C6900", "C6901", "C6902", "C6910", "C6911", "C6912", "C6920", "C6921", "C6922", "C6930", "C6931", "C6932", "C6940", "C6941", "C6942", "C6950", "C6951", "C6952", "C6960", "C6961", "C6962", "C6980", "C6981", "C6982", "C6990", "C6991", "C6992", "C700", "C701", "C709", "C710", "C711", "C712", "C713", "C714", "C715", "C716", "C717", "C718", "C719", "C720", "C721", "C7220", "C7221", "C7222", "C7230", "C7231", "C7232", "C7240", "C7241", "C7242", "C7250", "C7259", "C729", "C73", "C7400", "C7401", "C7402", "C7410", "C7411", "C7412", "C7490", "C7491", "C7492", "C750", "C751", "C752", "C753", "C754", "C755", "C758", "C759", "C760", "C761", "C762", "C763", "C7640", "C7641", "C7642", "C7650", "C7651", "C7652", "C768", "C7A00", "C7A010", "C7A011", "C7A012", "C7A019", "C7A020", "C7A021", "C7A022", "C7A023", "C7A024", "C7A025", "C7A026", "C7A029", "C7A090", "C7A091", "C7A092", "C7A093", "C7A094", "C7A095", "C7A096", "C7A098", "C7A1", "C7A8", "D469", "E3121", "E3122", "E3123" |

International Classification of Diseases, 10th revision (ICD-10).
